# Influence of Graphene Oxide Nanoparticles on Bond-Slip Reponses between Fiber and Geopolymer Mortar

**DOI:** 10.3390/nano12060943

**Published:** 2022-03-13

**Authors:** Darrakorn Intarabut, Piti Sukontasukkul, Tanakorn Phoo-ngernkham, Hexin Zhang, Doo-Yeol Yoo, Suchart Limkatanyu, Prinya Chindaprasirt

**Affiliations:** 1 Construction and Building Materials Research Center, Department of Civil Engineering, Faculty of Engineering, King Mongkut’s University of Technology North Bangkok, Bangkok 10800, Thailand; darrakorn.in@gmail.com or; 2 Sustainable Construction Material Technology Research Unit, Department of Civil Engineering, Faculty of Engineering and Architecture, Rajamangala University of Technology Isan, Nakhon Ratchasima 30000, Thailand; tanakorn.ph@rmuti.ac.th; 3 School of Engineering and the Built Environment, Edinburgh Napier University, Edinburgh EH14 1DJ, UK; j.zhang@napier.ac.uk; 4 Department of Architectural Engineering, Hanyang University, Seongdong-gu, Seoul 04763, Korea; dyyoo@hanyang.ac.kr; 5 Department of Civil Engineering, Faculty of Engineering, Hat Yai Campus, Prince of Songkla University, Songkla 90110, Thailand; suchart.l@psu.ac.th; 6 Sustainable Infrastructure Research and Development Center, Department of Civil Engineering, Faculty of Engineering, Khon Kaen University, Khon Kaen 40002, Thailand; prinya@kku.ac.th; 7 Academy of Science, Royal Society of Thailand, Dusit, Bangkok 10210, Thailand

**Keywords:** geopolymer, graphene oxide, single fiber pullout, bond-slip, rate sensitive

## Abstract

In this study, the influence of graphene oxide nanoparticles on the bond-slip behavior of fiber and fly-ash-based geopolymer paste was examined. Geopolymer paste incorporating a graphene oxide nanoparticle solution was cast in half briquetted specimens and embedded with a fiber. Three types of fiber were used: steel, polypropylene, and basalt. The pullout test was performed at two distinct speeds: 1 mm/s and 3 mm/s. The results showed that the addition of graphene oxide increased the compressive strength of the geopolymer by about 7%. The bond-slip responses of fibers embedded in the geopolymer mixed with graphene oxide exhibited higher peak stress and toughness compared to those embedded in a normal geopolymer. Each fiber type also showed a different mode of failure. Both steel and polypropylene fibers showed full bond-slip responses due to their high ductility. Basalt fiber, on the other hand, because of its brittleness, failed by fiber fracture mode and showed no slip in pullout responses. Both bond strength and toughness were found to be rate-sensitive. The sensitivity was higher in the graphene oxide/geopolymer than in the conventional geopolymer.

## 1. Introduction

Geopolymers are a type of cementitious material synthesized from raw materials containing a high content of aluminum and silicon [1,2,3,4,5,6,7,8,9]. Since the chemical reaction occurs in the polymerization process, it is usually called a geopolymer. The production of geopolymers requires no Portland cement, and the raw materials are often byproducts or waste materials that normally, if not being utilized, would find their way to landfill. Therefore, the production of geopolymers is not only sustainable in terms of reducing Portland cement usage but also in diverting waste from landfill.

Hardened geopolymer is known to have mechanical properties similar to hardened concrete produced from Portland cement. It usually exhibits excellent compressive strength but poor tensile strength, and to improve the brittleness, short fibers are randomly mixed with the geopolymer [10,11,12,13,14,15]. The effectiveness of fiber comes from its ability to bridge across cracks to prohibit or slow down crack propagation, which allows materials to carry load beyond the first cracking [16,17,18,19].

For fiber-reinforced cementitious material (FRC), the performance depends on factors such as fiber type, geometry, orientation, and volume fraction [20,21,22,23,24]. Additionally, the mechanical properties of the cementitious matrix also play a key role in the performance of FRC, whereby the improvement in mechanical properties of the matrix also has a positive effect on the mechanical properties of FRC [24,25]. In practice, the properties of a cementitious matrix can be enhanced using property enhancement additives. For example, phase change materials can be used to improve thermal storage [25,26,27,28,29,30,31], crumb rubber to improve energy absorption [32,33,34,35], or nanomaterials to improve mechanical properties [36,37,38,39,40], etc.

According to the aforementioned literature review, there are a large number of investigations on the effect of graphene oxide on properties of cement- and fiber-reinforced cementitious materials. However, not many studies have investigated the effect of graphene oxide on geopolymer, especially fiber-reinforced geopolymer. In this study, the effect of a nanomaterial, graphene oxide type, on the mechanical properties of fiber-reinforced geopolymer (FRG) was investigated. In general, the performance of FRC and FRG can be assessed through standardized tests such as ASTM C1609 [41], ASTM C1399 [42], or JSCE SF4 [43], which are based mainly on flexural testing. However, to investigate the fiber–matrix interaction, a so-called single fiber pullout test is more appropriate [40,44,45]. The graphene oxide (GO) solution was produced at a concentration of 10 mg/mL, and the incorporation rate of GO was 0.05% by weight of the binder. The specimens were prepared in a half-briquette form and two loading rates of 1 and 3 mm/s were carried out. The failure pattern, bond-slip response, strength, and toughness of the obtained results were discussed.

## 2. Experimental Process

### 2.1. Materials

The following materials were used to develop the FRG: binder, fine aggregate, alkaline activator, GO, and various fibers. The binder was a Class C fly ash (FA), according to ASTM C168, with a specific gravity of 2.61 and chemical composition as shown in Table 1. The fine aggregate was river sand (RS) with a specific gravity of 2.85 and particle size between sieve no. 4 and 16. The alkaline activator was used as a liquid solution of sodium hydroxide (SH, NaOH) and sodium silicate (SS, Na_2_SiO_3_). The concentration of SH was set constant at 12 Molars. The concentration of SS dissolved in water was 34.2%. The GO solution was dispersed in water at a concentration of 10 mg/mL; a modified Hummers technique was used to prepare the GO [46,47], and its properties are given in Table 2. The fibers used were steel (SF), polypropylene (PF), and basalt (BF) microfibers. Table 3 summarizes the properties of these fibers.

### 2.2. Preparation of Specimen

**Figure 1:**
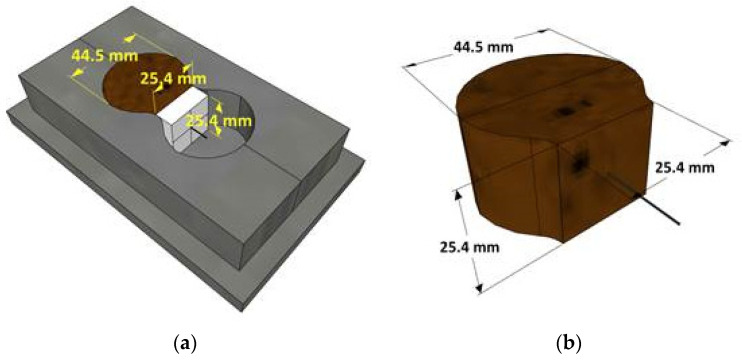
Specimen preparation (**a**) mold setup and (**b**) specimen after being demolded.

Specimens were produced in the shape of a half briquette with dimensions as given in Figure 1a. For the control geopolymer mortar (GM), sand to binder (s/b) ratio of 1.25, liquid to binder (l/b) ratio of 0.40, and SS/SH ratio of 1.0 were used. The mixing process began by dry mixing FA and RS for about 2 min. Then, SH solution was added and mixed for 1 min. After that, SS solution was added and then mixed for another minute. In the case of the graphene-modified geopolymer mortar (GOGM) sample, a similar process was employed, and the graphene oxide solution (GO) at 0.05% by weight of FA was mixed with SH prior to the beginning of the mixing process. In order to ensure the uniform distribution of GO, the GO solution was dispersed in the SH solution and blended for 20–30 s until the GO was fully distributed in the solution. The SH+GO solution was then poured into a dry base (sand and fly ash) and mixed for 1 min. The SS solution was subsequently added, and the mixing continued for 1 min. The mix proportions and corresponding compressive strengths are given in Table 4.

The specimens were prepared in half briquette form using a piece of Styrofoam to block half of the briquette mold and to hold a fiber at the center, as shown in Figure 1b. Before casting, one end of the fiber was inserted through a hole in the middle of the foam to be embedded in the geopolymer mortar. The other end, with a length of 20 mm, was exposed on the empty side. The mortar was then poured into the mold and consolidated on a vibrating table for 10 s. The cast specimens were covered with a plastic sheet to prevent moisture loss. After 24 h, the specimens were demolded, wrapped in a plastic sheet, and kept in a controlled temperature room at 25 °C until the date of the test (28 days). Three different fibers and two different mortar types were used to produce the specimens, as listed in Table 5.

### 2.3. Experimental Program

**Figure 2:**
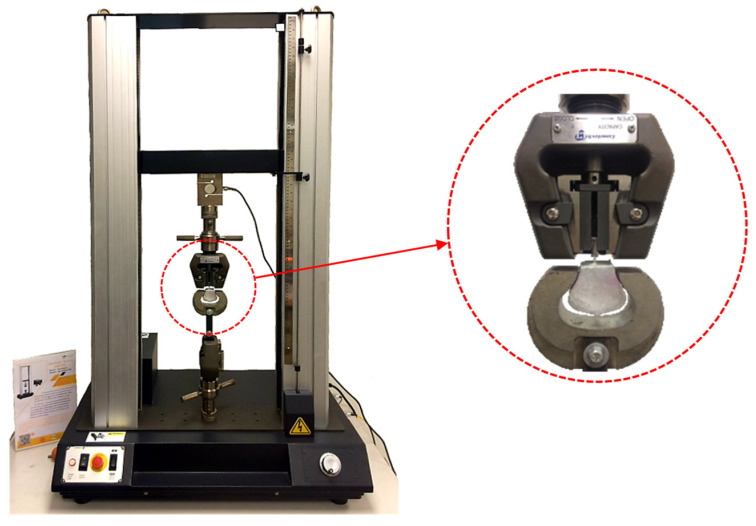
Single fiber pullout test setup.

The compressive strength of geopolymer mortar (both GM and GOGM) was tested after 7 and 28 days, in accordance with ASTM C109 [48]. The single fiber pullout test was carried out using a 10 kN UTM machine (Instron (Thailand) Co., Ltd., Bangkok, Thailand), as illustrated in Figure 2. The effect of the loading rate was examined using two different loading rates (i.e., 1 and 3 mm/s). An average of three samples were used to represent the test results.

Using the results from the single fiber pullout test (i.e., axial force and slipping distance), the axial tensile stress of fiber (*σ_f_*), average interfacial bond stress (*τ_av_*), and energy absorption (*E*, toughness) can be calculated using Equations (1)–(3) as follows:

(1) $$\sigma_{f}=\frac{P}{A_{f}},$$

(2) $$\tau_{av}=\frac{P}{\pi d_{f}L},$$

(3) $$E=\int_{0}^{\delta }Pd\delta ,$$

where *P* = pullout force (N), *A_f_* = cross sectional area of the fiber (mm^2^), *L* = embedded length (mm), *d_f_* = fiber diameter (mm), and *δ* = fiber slipping distance (mm).

## 3. Result and Discussion

### 3.1. Compressive Strength

**Figure 3:**
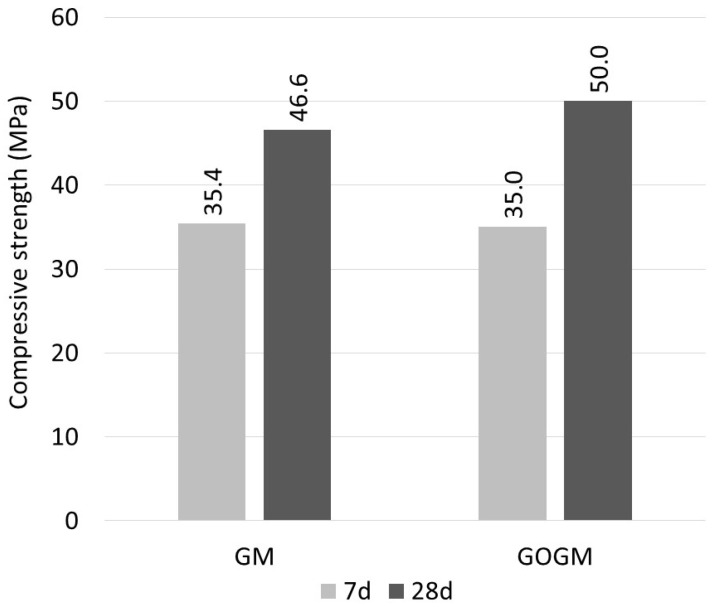
Compressive strength of geopolymer mortar.

The compressive strength results of the GM and GOGM after 7 and 28 days are shown in Figure 3. The results after 7 days yielded strengths about 70–75% of those after 28 days. The GO added at the 0.05 wt% of FA dosage insignificantly affected the 7-day compressive strength. However, it slightly increased the 28-day strength by about 8% due to the effect of GO promoting geopolymerization. Similar findings have also been reported, for example, by Xu et al. [49], who showed that GO improved the polymerization degree of FA geopolymer. Ranjbar et al. [50] found that the addition of GO increased energy absorption during pullout, which led to an improvement in toughness. Saafi et al. [51] stated that GO changed the morphology of geopolymers from a porous nature to a considerably pore-filled morphology, which led to an increase in compressive strength. The addition of nanoparticles such as GO can reduce the porosity of the geopolymer with an increase in geopolymer gel [52].

### 3.2. Energy Dispersive X-ray Spectroscopy (EDS)

The EDS performed on both the GM and GOGM samples are given in Table 6. Energy-dispersive X-ray spectroscopy (EDS, EDX, EDXS or XEDS), sometimes called energy-dispersive X-ray analysis (EDXA or EDAX) or energy-dispersive X-ray microanalysis (EDXMA), is an analytical technique used for the elemental analysis or chemical characterization of a sample. The increase in carbon content (C) from 1.19 to 2.18% indicated the existence of GO in the geopolymer. The geopolymerization reactions that formed N-A-S-H and Si-O-Al products were also promoted by the addition of GO, as seen by the increasing weight of Si from 13.51 to 14.72% and Na from 12.00 to 13.41% [52,53].

### 3.3. Fiber Pullout

#### 3.3.1. Failure Pattern

**Figure 4:**
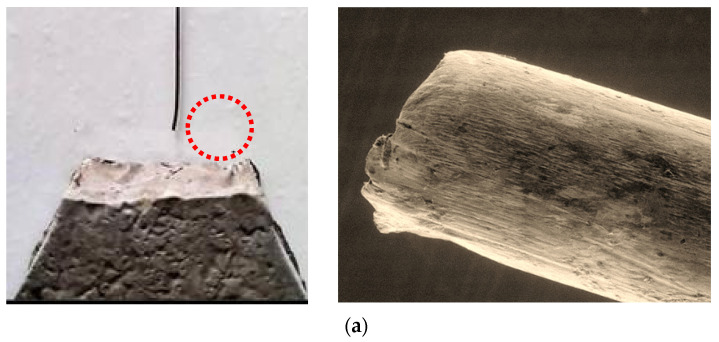

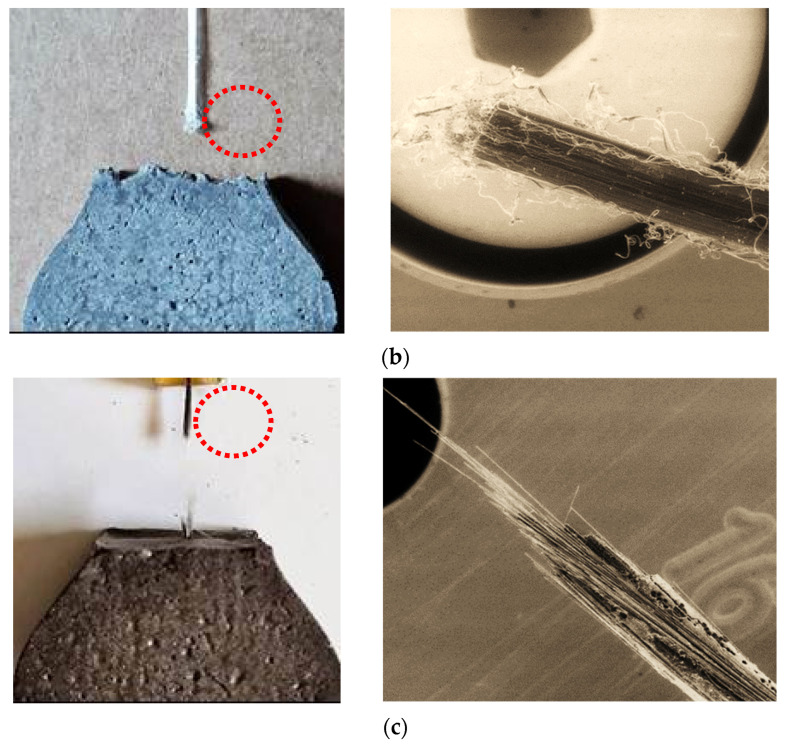
SEM images of fiber after being pulled out: (**a**) SF, (**b**) PF, and (**c**) BF.

Two failure patterns were observed in this study, i.e., fiber slipping and fiber fracture. The failure mode depended on the fiber tensile strength and bond stress between the fiber and matrix. If the fiber tensile strength was greater than the applied bond stress, the fiber slipped and was completely pulled out from the mortar without fiber fracture or breakage. This was found in the case of SF and PF (Figure 4a,b). In contrast to fiber slipping, when the bond strength was greater than the fiber tensile strength, fibers were broken before being completely pulled out from the mortar. This failure mode is known as fiber fracture, which was observed in the BF fiber (Figure 4c).

Figure 4 illustrates the failure mode of each fiber type at the microscopic level. For SF, the deformed (hooked) part at the fiber’s end (deformed end) appeared to be straightened before being pulled out entirely from the mortar, but no major damage to the fiber end was seen (see Figure 4a). For PF, the fiber’s surface appeared to have been scraped against the interface between the mortar and fiber during the pullout process, as seen by the occurrence of microfiber strands being scraped out along the surface. At the end of the PF fiber, severe damage was observed, with large amounts of fiber strands accumulating there (Figure 4b). It was noted that, although the hooked end of SF greatly increased bond strength via mechanical anchorage, its tensile strength was much greater than the bond stress. Although the PF tensile strength was considerably lower than that of SF, it had no deformed part to create mechanical bond stress; therefore, its bonding to mortar was even less. Hence, both SF and PF slipped without breaking.

For BF, since it is a twisted bundle-type fiber with a coating material, partial debonding of the coating material throughout the fiber length could be noticed during the pullout process (Figure 4c). Additionally, for a bundle-type fiber, the small spaces between each fiber strand cause the fiber to have high absorption. This allows the geopolymer paste to be absorbed along the fiber surface, which significantly improves the interfacial bonding between the fiber and matrix. In the case of BF, the improvement in interfacial bonding was up to the point where the interfacial bond stress became higher than the fiber strength and caused the fiber to fail in fracture mode (Figure 4c). At the tip of the fiber end, uneven tearing and end splitting of small BF fiber strands were observed, which indicated that the fiber suffered great stress during the pullout process. Similar findings were reported by Chindaprasirt et al. [40], where bundle-type glass fiber was found to exhibit better bonding and a strong interfacial bond.

#### 3.3.2. Single Fiber Pullout Response

**Figure 5:**
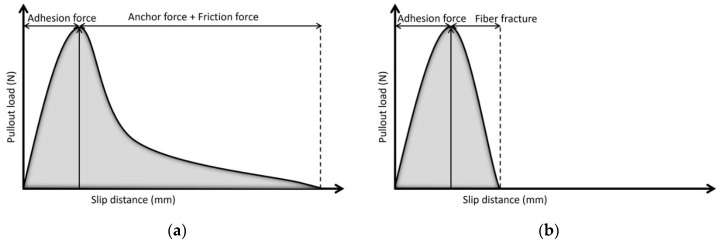
Typical bond-slip response of: (**a**) fiber pullout and (**b**) fiber fracture.

Figure 5 schematically illustrates the typical bond-slip behaviors of the single fiber pullout test. The fiber pullout behavior can be categorized into pre-peak and post-peak. Initially, the entire embedded fiber is bonded or adhered to the mortar. When a fiber is subject to a pullout load, the load is transmitted to the mortar through adhesion force. During the initial linear response, the adhesion force developed corresponding to the applied load to keep the fiber in resting condition. Once the load reaches its peak, the fiber either fractures or begins to lose adhesion and slip.

In the case where the ultimate strength of the fiber is greater than the peak stress, the fiber is not fractured but starts to slip out of the mortar (Figure 5a). As the fiber slips, two main forces, i.e., friction force (taking place as fiber moves against mortar) and anchorage force (obtained from any mechanical anchorage and fiber geometry), govern the load carried in the post-peak response.

In the case where the ultimate strength of the fiber is lower than the peak stress, as soon as the pullout load reaches the peak, the fiber begins to fracture, and the load quickly drops to zero simultaneously (Figure 5b).

##### Effect of Graphene Oxide

**Figure 6:**
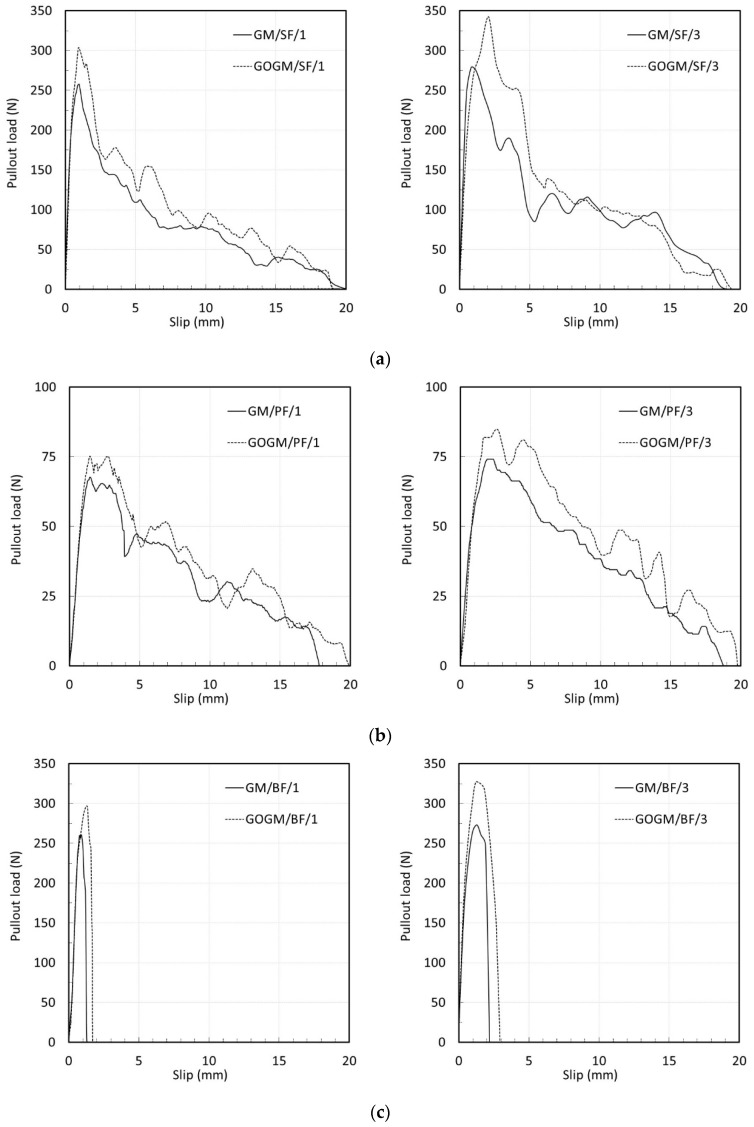
Effect of graphene oxide on bond-slip responses of: (**a**) SF, (**b**) PF, and (**c**) BF.

Figure 6a–c demonstrate the effect of GO on the bond-slip response of SF, PF, and BF subjected to 1 and 3 mm/s loading rates. The bond-slip responses of SF embedded in GM and GOGM are shown in Figure 6a. Prior to the peak, a linear bond-slip response was observed. The deformation of the steel fibers increased linearly with the increasing applied load. Once the load reached its peak, the fiber began to slip, followed by a large drop in load. Due to the smooth and hard surface of the steel fiber, the interface bond was not particularly strong. However, as the fiber slipped out, the rate the load was dropping began to slow down. The slow descending post-peak response can be mainly attributed to the deformed shape (hooked end) at end of the fiber. As the fiber began to be pulled out of its resting place, the hooked end provided resistance to the pulling force. Once the hook was forced to be straightened, the fiber was pulled out easily, and the load then decreased to zero. The addition of GO appeared to increase the peak pulling load. In addition to the anchorage force, the stronger geopolymer matrix provided additional resistance to the pullout force. The increase in peak load in the case of SF was observed at around 7% and 12% for a loading rate of 1 and 3 mm/s, respectively (Table 7 and Table 8).

Figure 6b shows the bond-slip behavior of PF. Similar to the case with SF, the load increased linearly with the fiber deformation prior to the peak load. However, because of the highly elastic and low strength characteristics of the PF, larger deformations at the peak load and smaller peak loads were observed compared to SF. Beyond peak load, a gradual decrease in pullout load was partly due to the friction bond between the fiber surface and geopolymer matrix. Another possible factor could have been the accumulation of scraped tiny fiber strands along the surface and at the fiber end, which provided resistance to the pulling load and helped to slow the rate of load drop. The post-peak response of PF varied from SF in that there was no large drop of load after the load reached its peak. This indicated that PF did bond better to the geopolymer paste than SF due to its rough and wavy surface. Immediately after the peak load, the load gradually decreased to zero. As seen in Figure 6b, the SEM image of a completely pulled out PF showed evidence of scraped fiber strands accumulating at the fiber tip. GO seemed to influence the peak pulling force of PF in the range of 11 to 14% for loading rates 1 and 3 mm/s, respectively (Table 7 and Table 8).

Figure 6c shows the bond-slip responses of BF. In the case where fiber fracture occurred, the responses were short and brittle. As soon as the load reached its peak, fiber fracture occurred, and the load fell instantaneously. The effect of GO caused the peak load to increase from 13.8 to 19.7%—the highest among the three fibers (Table 7 and Table 8). This is partly because of the improvement in bond strength of the BF due to its high absorbability.

It must be noted here that even though the BF failed in fiber fracture mode, the axial tensile stress at peak load was lower than the fiber tensile strength capacity (900 MPa) (Table 7 and Table 8). This can be explained as follows. Since the BF are a twisted bundle-type fiber, this means that the small fiber strands are, in fact, embedded in the paste at a slightly inclined angle. This can lead to an increase in bridging force, which creates higher stresses in the fiber due to both bending and axial forces [54,55]. Choi et al. [56] reported that the tensile strength of BF decreased with an increase in the inclined angle of the fiber.

##### Effect of Loading Rate on Bond-Slip Response

**Figure 7:**
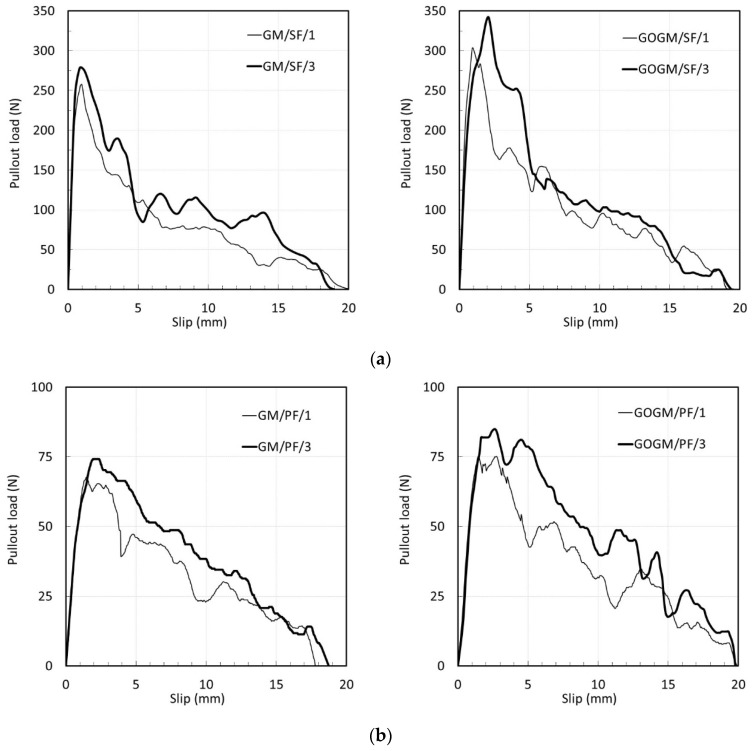

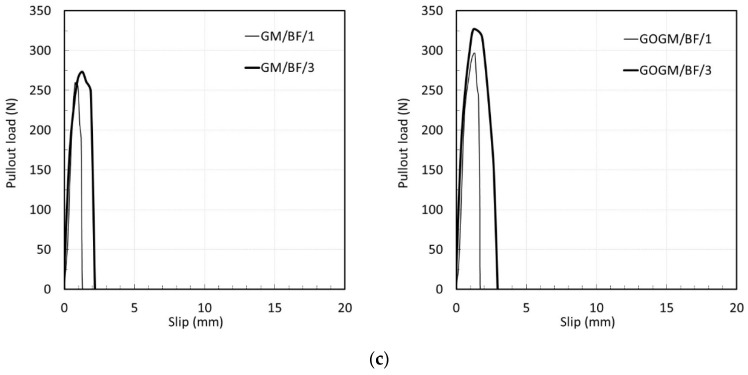
Effect of loading rate on bond-slip responses of: (**a**) SF, (**b**) PF, and (**c**) BF.

Figure 7a–c show the effect of loading rate on the bond-slip responses. Regardless of fiber and mortar type, the increase in loading rate caused the peak load to increase. The results given in Table 7 and Table 8 indicate an increase of about 8.0, 9.6, and 4.8% in GM/SF, GM/PF, and GM/BF, respectively. Theoretically, the mechanical properties of composite materials such as fiber-reinforced cementitious materials are known to be sensitive to the loading rate, meaning that they increase as the rate of loading increases [57,58,59,60,61,62,63,64]. This is also the case for the single-pullout test. Babafemi [65] reported an increase in the pullout load of macro synthetic fiber with the increasing rate of loading and embedment length. Bindiganavile [66] found an increase in peak pullout load and a change in the mode of failure as the load scheme changed from quasi-static to impact pullout load. Comparing the three fibers, BF is less sensitive than both SF and PF, which is perhaps due to the fracture mode failure of BF.

In the case of GOGM, increases of about 12.7, 12.9, and 10.2% were observed for GOGM/SF, GOGM/PF, and GOGM/BF, respectively. The GOGM appears to be more sensitive to loading rate than GM, as seen by the higher increasing percentage. This contributes mainly to the higher compressive strength of GOGM due to the addition of GO. Kim et al. [67] similarly reported the increase in rate sensitivity of fiber embedded in cement material with higher compressive strength.

#### 3.3.3. Mechanical Properties

**Figure 8:**
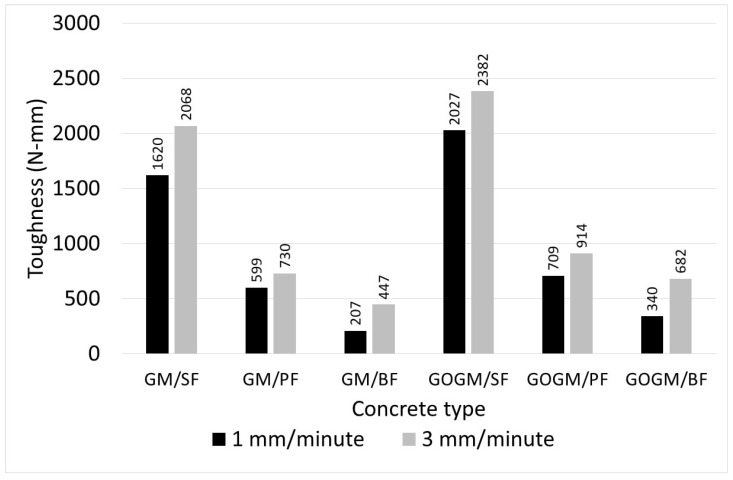
Pullout energy or toughness.

The results of peak pullout load, strength, and bond strength are given in Table 7 and Table 8, and the pullout energy (toughness) is shown in Figure 8. Both peak pullout stress and bond strength in GOGM (SF/GOGM, PF/GOGM, and BF/GOGM) were higher than in GM (SF/GM, PF/GM, and BF/GM). They were increased by 11–17% for a loading rate of 1 mm/s and 14–23% for a loading rate of 3 mm/s.

For bond-slip toughness, the SF provided the highest toughness among the fibers (around 1620–2382 N-mm) due to its high strength and ductility. The PF, which has relatively lower strength, produced lesser toughness (around 599–914 N-mm). Even though BF has higher tensile strength than PF, its brittleness and twisted bundle alignment caused the fracture failure to occur prior to slipping. This cut short its bond-slip responses and prevented a post-peak response from occurring. Thus, BF yielded the lowest toughness (around 207–682 N-mm). Regarding the effect of GO, the toughness was found to increase at different angles depending on the fiber type.

**Figure 9:**
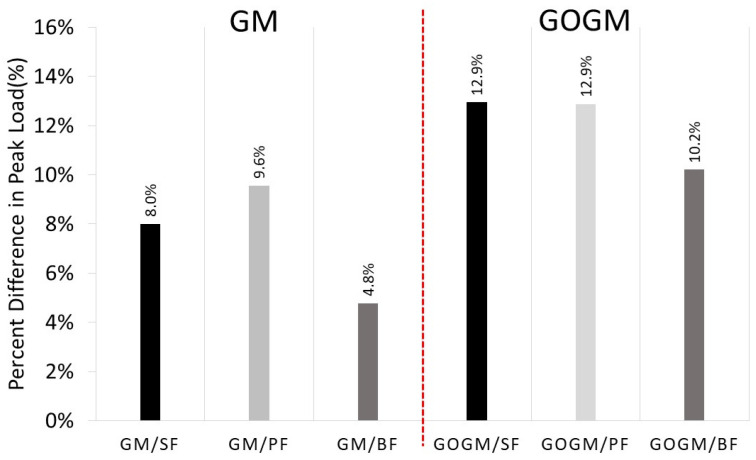
Percentage difference in peak load due to increasing loading rate comparing between GM and GOGM.

Comparing GM and GOGM, the percentage difference in peak load at two different rates of loading is shown in Figure 9. For GM, the increase in peak load was found at around 8, 9.6, and 4.8% for GM/SF, GM/PF, and GM/BF, respectively. For GOGM, the increase in peak load was observed at around 12.9, 12.9, and 11.2% for GOGM/SF, GOGM/PF, and GOGM/BF, respectively. This indicated that the GOGM is more sensitive to the rate of loading than GO. This perhaps has to do with the increase in matrix strength, which provided more resistance to the pull force.

## 4. Conclusions

The addition of graphene oxide at a dosage of 0.05% weight of binder (fly ash) increased the compressive strength of GM by about 7.3%.

From the single fiber pullout test, the failure mode of SF and PF was fiber slipping mode, whereas for BF, it was fiber fracture mode. The SEM revealed that the surface of PF was being scraped against the matrix, which caused the accumulation of tiny fiber strands at the fiber tip. For BF, the fracture of individual fibers was observed. There were signs of fiber being tortured and fractured unevenly.

For the pullout response, because of their high ductility, both SF and PF showed full bond-slip responses. The effect of GO increased the bond strength and toughness of both fibers, though at different degrees depending on fiber type. For BF, the response was brittle, and there was no fiber slip because the fiber fractured before slipping out from the matrix. The effect of GO also yielded higher peak bond stress and toughness.

For the effect of the loading rate, both bond strength and toughness were found to be dependent on the loading rate. Increasing the loading rate from 1 to 3 mm/s caused the peak bond stress to increase by about 5–10% for GM and 10–12% for GOGM and the toughness to increase by 6–116% for GM and 19–46% for GOGM.

## Figures and Tables

**Table 1:** Chemical compositions of fly ash.

Materials	SiO_2_	Al_2_O_3_	Fe_2_O_3_	CaO	MgO	Na_2_O	K_2_O	SO_3_	LOI
FA (%)	31.85	15.89	14.07	26.76	3.66	1.95	1.95	2.45	0.17

**Table 2:** Properties of graphene oxide solution.

Graphene Oxide Solution (GO)	
Appearance	Brown/Black
Solvent	Dispersion
Concentration (mg/mL)	10

**Table 3:** Properties of fibers.

Category	SF	PF	BF
Shape	Hooked end	Crimped	Twisted bundle
Length (mm)	35	55	43
Diameter (mm)	0.55	0.85	0.72
Tensile strength (MPa)	1345	365	900
	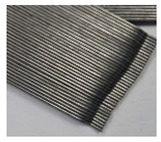	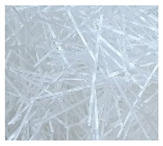	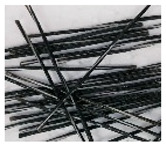

**Table 4:** Mix proportion and compressive strength.

No.	Symbols	FA (kg/m^3^)	RS (kg/m^3^)	SS (kg/m^3^)	SH (kg/m^3^)	GO (%*w*/*w* of FA)
1	GM	879	1099	176	176	0
2	GOGM	879	1099	176	176	0.05%

**Table 5:** Casting schedule.

Symbol	Type	Fiber	Number of Specimen per Loading Rate	
			1 mm/s	3 mm/s
GM/SF	Geopolymer mortar (GM)	Steel	3	3
GM/PF		Polypropylene	3	3
GM/BF		Basalt	3	3
GOGM/SF	Geopolymer mortar with graphene oxide (GOGM)	Steel	3	3
GOGM/PF		Polypropylene	3	3
GOGM/BF		Basalt	3	3

**Table 6:** Elemental analysis with EDS.

Element	Weight (%)		Atomic (%)	
	GM	GOGM	GM	GOGM
C	1.19	2.18	1.97	3.66
O	53.44	51.03	66.61	64.07
Na	12.00	13.14	10.41	11.54
Mg	1.04	1.02	0.89	0.83
Al	5.73	6.59	4.24	4.88
Si	13.51	14.72	9.60	9.41
S	2.02	1.59	1.26	1.00
Ca	11.10	12.76	5.57	6.48
Fe	3.51	3.23	1.33	1.19

**Table 7:** Peak load and strength of fiber subjected 1 mm/s loading rate.

Specimen Type	Peak Load (N)	Slip at Peak Load (mm)	Tensile Strength (MPa)	Avg. Bond Strength (MPa)	S.D. (MPa)
GM/SF/1	258	1.00	1085	7.46	0.07
GM/PF/1	68	1.47	119	1.27	0.04
GM/BF/1	261	0.88	641	5.77	0.08
GOGM/SF/1	303	0.94	1276	8.77	0.13
GOGM/PF/1	75	1.46	132	1.41	0.03
GOGM/BF/1	297	1.30	729	6.56	0.10

**Table 8:** Peak load and strength of fiber subjected 3 mm/s loading rate.

Specimen Type	Peak Load (N)	Slip at Peak Load (mm)	Tensile Strength (MPa)	Avg. Bond Strength (MPa)	S.D. (MPa)
GM/SF/3	278	0.83	1171	8.05	0.09
GM/PF/3	74	1.92	131	1.39	0.04
GM/BF/3	273	1.28	671	6.04	0.08
GOGM/SF/3	342	2.06	1441	9.91	0.09
GOGM/PF/3	85	2.55	149	1.59	0.09
GOGM/BF/3	327	1.23	803	7.23	0.11

## Data Availability

Not applicable.
